# The reliability and usability of the Anesthesiologists’ Non-Technical Skills (ANTS) system in simulation research

**DOI:** 10.1186/s41077-016-0013-2

**Published:** 2016-06-08

**Authors:** Laura Zwaan, Lian Tjon Soei Len, Cordula Wagner, Dick van Groeningen, Mark Kolenbrander, Ralf Krage

**Affiliations:** 1 Department of Public and Occupational Health, EMGO Institute for Health and Care Research, VU University Medical Center, Amsterdam, The Netherlands; 2 Department of Anaesthesiology, VU University Medical Center, Amsterdam, The Netherlands; 3 NIVEL, Netherlands Institute for Health Services research, Utrecht, The Netherlands; 4 Department of Anaesthesiology, Utrecht University Medical Center, Utrecht, The Netherlands; 5 Institute of Medical Education research Rotterdam (iMERR), Erasmus MC, Rotterdam, The Netherlands

**Keywords:** Interrater Reliability, Situation Awareness, Chest Compression, Intraclass Correlation Coefficient, Distractor Condition

## Abstract

**Background:**

Non-technical skills (NTS) such as leadership and team work are important in providing good quality of care. One system to assess physicians’ NTS is the Anesthesiologists’ Non-Technical Skills (ANTS) system. The present study evaluates the ANTS system on the interrater reliability and usability for research purposes.

**Methods:**

Ten anesthesiologists and 20 anesthesiology residents performed two resuscitation scenarios (with and without the presence of distractors) in a simulation room with a full-scale patient simulator. The scenarios were videotaped. Two independent raters rated the NTS of the anesthesiologists using the ANTS system. The intraclass correlation coefficients (ICC) were calculated to determine the interrater reliability of both the total NTS score and the measured differences between the two scenarios. The raters filled out a questionnaire to obtain insights in the usability of the ANTS system for research purposes.

**Results:**

The ICC for the total score of the NTS was substantial (0.683), and the ICC of the elements varied between 0.371 for assessing capabilities and 0.670 for providing and maintaining standards. The intraclass correlation coefficient of measuring differences was fair (0.502). The raters judged the usability as good.

**Conclusions:**

The ANTS system was reliable for the total score and usable to measure physicians’ NTS in a research setting. However, there was variation between the reliability of the elements. We recommend that if the ANTS is used for research, a pilot study should determine elements not applicable or observable in the scenario of interest; these elements should be excluded from the study.

**Electronic supplementary material:**

The online version of this article (doi:10.1186/s41077-016-0013-2) contains supplementary material, which is available to authorized users.

## Background

Anesthesiologists work in a high-risk environment, in which the workload highly fluctuates. Besides technical skills, non-technical skills (NTS) such as teamwork and leadership are important to be able to perform well in stressful situations [1, 2]. NTS are a combination of cognitive and social skills, which complement knowledge and technical skills and contribute to physicians’ performance [1, 3]. While technical skills have always been the core of medical education, NTS have recently emerged in the medical education programs as well [1, 2, 4–6].

Several rating systems have been developed to evaluate the NTS, which particularly focus on surgeons and anesthesiologists [7–11]. Most of the rating systems assessing NTS are behavioral marker systems and assess elements of NTS such as Task Management (e.g., Planning, Preparing, and Prioritizing) and Situation Awareness (e.g., Anticipating). The “Anaesthetists’ Non-Technical Skills” (ANTS) system assesses the NTS of anesthesiologists and is developed for educational purposes [7, 12, 13]. Generally, after a training session, the elements of the ANTS system are rated and discussed with the observed anesthesiologists, which helps them to improve their NTS. Since an increasing number of studies have shown the importance of NTS, the ANTS can also be considered as an important measure to assess performance in research aimed at improving NTS. The psychometric properties, such as the usability and reliability of the ANTS, have been assessed by the developers of the ANTS system and were considered to be of acceptable level [14]. A recent study assessed the reliability after a 1-day training for raters and concluded that the reliability was poor [15]. However, when the ANTS system is used for research purposes, more extensive training of the observers is necessary. Specifically, compared to the use of the ANTS in clinical settings, in research settings, it is important to be able to compare participants with each other, and therefore, the same set of elements should be rated for all participants. Additionally, for research, not only a reliable score is important, but the ability of an instrument to reliably identify differences between research conditions (i.e., between experimental and control conditions) is essential as well. The aim of this study is to determine the interrater reliability of the ANTS system, the interrater reliability of measuring differences between experimental conditions and the usability of the ANTS system when used for research purposes.

## Methods

The study was conducted at the clinical simulation center of the VU University Medical Center in Amsterdam, the Netherlands. Each participant in this randomized cross-over study participated in two simulated resuscitation scenarios, which were videotaped. One “standard” resuscitation scenario without external distractors served as control condition, and the experimental condition involved a scenario with additional distractors (background noise and the presence of a family member). The NTS of physicians during the resuscitations were assessed by two raters, who rated the performance using the ANTS system. The reliability was determined using the intraclass correlation coefficient (ICC). The usability was determined by a short questionnaire filled out by the raters which, among other things, assessed the observability and difficulty of the ANTS system.

### Observed participants and raters

Thirty physicians were observed in the study. They were all part of the hospital resuscitation team and trained in advanced life support. All participants were employed at the VU University Medical Center in Amsterdam, the Netherlands. Of the participants, 17 were male and 13 female, their average age was 35 years (SD = 4.7), and they had on average 6.6 (SD = 3.6) years of work experience as a physician. Out of the 30 participants, 26 had been in the simulator before for educational purposes. All participants signed the informed consent form and granted approval to the research team to analyze the videos.

There were two raters who both scored all 60 videos. One rater (male) was an experienced anesthesiology nurse and a medical student. The second rater was a (female) research psychologist with a focus on patient safety. Both raters had attended simulation sessions with resuscitation scenarios prior to rating the videos and were aware of the research question.

### Experimental setting

#### Simulator

The simulator room was designed as a shock room and equipped with a full-scale patient simulator (SimMan^™^, Laerdal Medical Corporation, Stavanger, Norway), on which all necessary tasks could be performed, i.e., chest compressions, defibrillation, administering medication, checking the pulse and carotid artery, etc.

Three video cameras from different positions recorded the sessions.

#### Procedure

The participants were welcomed and provided with information about the study. Subsequently, the first scenario was explained and they entered the simulator room. We counterbalanced the order of the scenarios to correct for the learning effect. Half of the participants were randomly selected to start with the scenario with additional distractors, and half the participants started with the scenario without distractors.

In both scenarios, a resuscitation scenario was performed, either a ventricular fibrillation (VF) or a ventricular tachycardia (VT). In both scenarios, the participants were assigned the role of team leader (which is the role anesthetists have in clinical practice) and were provided with three additional team members: a first-year anesthesia resident, a medical student, and an emergency room nurse. The team members were part of the research group and were instructed to perform medical acts such as chest compressions, defibrillation, and medication preparation, only on request of the participant anesthetist. This allowed for the anesthetist participants to use their NTS and for the raters to only rate the NTS of the participant and not those of the other team members. The participants were instructed about the clinical context, the team members, and the simulator, but did not obtain instructions regarding NTS. The measurements for the study purposes ended after 8 min. After the first scenario ended, the participants had a 5-min break after which the second scenario started. No feedback was provided to the participant between the sessions, because everyone at the department frequently participates in simulation sessions for educational purposes, which contain extensive debriefing.

### ANTS system

The ANTS system is developed by the Industrial Psychology Research Center and the Scottish Clinical Simulation Center at the University of Aberdeen. The ANTS system is a behavioral marker system which assesses the NTS of anesthesiologists [7]. The NTS are divided into four categories: Task Management, Team Working, Situation Awareness, and Decision Making. Each of the categories has three to five underlying elements that more specifically describe the NTS (see Table 1). Each of the elements is described with a list of examples of poor and good behaviors which can support raters in identifying whether the NTS are present or absent.

**Table 1:** Examples of poor and good behaviors for each of the elements (categories and elements are adopted from the ANTS) [13]

Category	Element	Poor behavioral marker	Good behavioral marker
Task Management	Planning and Preparing	Starting to intubate the patient, while the intubation materials are not prepared	Timely preparation of medication (e.g., Amiodarone) before needing to administer it
	Prioritizing	Actively attending to the patients family member during complicated tasks	Mentioning the order in which tasks need to be performed (i.e., after defibrillation, I would like to intubate the patient)
	Providing and Maintaining Standards	Not checking whether anyone touches the bed while defibrillating	Double check of medication
	Identifying and Utilizing Resources	Not checking the capabilities of the co-workers and therefore not making use of their skills	Call for the resuscitation team
Team Working	Coordinating Activities with Team	Not giving specific orders to one person, but giving several tasks to the whole team in general	Asking the team members who they are, e.g., are you the intern?
	Exchanging Information	Starting with tasks without explaining what s/he is doing and why s/he is doing the task	Mentioning the patients status, e.g., we need to resuscitate the patient
	Using Authority and Assertiveness	Forgets to give team members tasks and tries to perform all the tasks him/herself	Clearly indicates the next steps and who needs to perform which tasks, e.g., if we still do not have a sinus rhythm after defibrillation, I want you to prepare 300 mg adrenaline.
	Assessing Capabilities	Gives orders to the intern, without checking whether s/he has sufficient knowledge to perform the task correctly	Asking the intern whether s/he knows how to give basic life support?
	Supporting Others	Negative or defensive tone when answering the team members’ questions	Complimenting team members
Situation Awareness	Gathering Information	Actively conducting tasks while not paying attention to the patient’s situation	Regularly checking the monitor, asking team members what they know about the patient
	Recognizing and Understanding	Not noticing that the patient is intubated incorrectly	Immediately recognizing that the patient needs to be resuscitated
	Anticipating	Not preparing for potential problems or possible next steps	Mentioning the next steps, e.g., in 2 min, we will defibrillate again; until then, I would like you to call the on-call cardiologist.
Decision Making	Identifying Options	Not mentioning options when decisions need to be made	Considering reasons why the patient has a VT/VF
	Balancing Risks and Selecting Options	No verbal considerations about the options and risks of the options	Initiates discussion on what to do next
	Re-evaluating	Does not show any verbal re-evaluation of the situation	Evaluates the situation and considers treatment options

Each of the elements is rated on a four-point scale: 1 = poor and means that the skills could not be observed in the scenario; 2 = marginal and signifies that the performance indicated cause for concern, considerable improvement is needed; 3 = acceptable meaning that performance was of a satisfactory standard but could be improved; while a score of 4 represents performance of a consistently high standard, enhancing patient safety, and could be used as a positive example for others [7]. The sum of the scores on the elements represents the total score of the categories. The possible scores for the category Task Management ranges from 4 to 12, Situation Awareness and Decision Making from 3 to 9, and Team Working from 5 to 20. The scores of all elements together represent the total NTS score, which ranges from 15 to 60. A score of 15 is obtained when all elements are rated as “poor,” while a total score of 60 means that all elements are rated as “good.”

### NTS rating procedure

The raters rated all 60 videos independently of each other. Prior to rating the videos with the ANTS system, the raters read the ANTS handbook and several articles about NTS [16]. Several practice sessions were conducted during which practice videos were rated and discussed in order to reach consensus on how to score the different elements of the ANTS form. For each of the elements of the ANTS, a list with typical examples of good practice and poor practice was developed specifically for the resuscitation scenario that was used in this study (see Table 1). This list was based on the good and poor behaviors described in the ANTS handbook but specified for the specific resuscitation scenarios. Furthermore, prior to rating the videos, the raters participated in a training session with a group of experts (including Dr. Rhona Flin). During this meeting, the use of the ANTS rating form was discussed for 2 h. For example, identifiers to differentiate between certain elements were discussed. This was followed by 2–3 h of practice with rating practice videos (not the videos used in this study). Everyone rated the elements of the ANTS for the videos independently of each other, and the scores were discussed among the participants. This contributed to the development of examples for good and poor behaviors.

Rating the videos included in this reliability study started with watching the complete video while making notes of good and poor behaviors. Subsequently, the video was watched again, during which the video was frequently paused to rate the elements of the NTS according to the ANTS system. In most cases, the video was watched three times in order to rate all elements of the ANTS.

### Usability measures

After all 60 videos were rated, the two raters who participated in this study both filled out a questionnaire on the usability of the ANTS system (Additional file 1). Some general questions on the completeness of the ANTS system were asked which involved all applicable questions of the questionnaire described in a study of the developers of ANTS [14]. Furthermore, the observability and difficulty of the different elements of the ANTS system were assessed.

### Statistical analyses

The ratings of all 60 videos of both reviewers were compared. The absolute agreement was calculated for all of the elements. The intraclass correlation coefficient (ICC) (Shrout and Fleiss convention ICC 3.1 agreement) was determined to provide information on the interrater reliability of the two raters. ICC agreement was calculated for the average measures for the NTS sum score, the four categories, and the individual elements.

The ICC was determined to obtain insight into the interrater reliability of measuring differences between scenarios. We calculated the ICC agreement score for average measures.

To analyze the usability questionnaire, descriptive statistics were used (SPSS Statistics for Windows, Version 20.0 (Armonk, NY: IBM Corp)).

## Results

### Reliability

The average total NTS score across all participants for rater 1 was 42.0 (SD = 5.6) and for rater 2 was 45.4 (SD = 4.5).

The overall ICC agreement for the sum score for evaluation of the videos was substantial, 0.683 (95 % CI: 0.247–0.845) [17]. The ICC agreement scores for the categories varied between 0.427 for Decision Making and 0.713 for Task Management. The ICC for the individual elements varied between 0.371 for assessing capabilities and 0.670 for providing and maintaining standards (see Table 2).

**Table 2:** Interrater reliability measures for the categories and elements of the ANTS system

Categories	ICC agreement	Elements	% absolute agreement	ICC agreement	Mean absolute difference
Task Management	0.713	Planning and Preparing	57	0.488	0.47
		Prioritizing	52	0.621	0.55
		Providing and Maintaining Standards	63	0.670	0.37
		Identifying and Utilizing Resources	62	0.449	0.45
Team Working	0.520	Coordinating Activities with Team	73	0.661	0.27
		Exchanging Information	57	0.398	0.52
		Using Authority and Assertiveness	46	0.519	0.55
		Assessing Capabilities	42	0.371	0.63
		Supporting Others	40	0.520	0.67
Situation Awareness	0.568	Gathering Information	55	0.409	0.45
		Recognizing and Understanding	50	0.535	0.53
		Anticipating	63	0.568	0.40
Decision Making	0.427	Identifying Options	32	0.459	0.85
		Balancing Risks and Selecting Options	33	0.426	0.80
		Re-evaluating	53	0.506	0.52

### The reliability of measuring differences between scenarios

Both raters had significant higher average score in the non-distractor condition compared to the distractor condition. For rater 1, the average scores for the non-distractor versus the distractor condition were 44.6 (SD = 0.886) versus 39.3 (SD = 9.06), *p* < 0.01, respectively. For rater 2, the average scores for the non-distractor versus the distractor condition were 46.5 (SD = 0.759) versus 44.0 (SD = 0.890), *p* < 0.05, respectively. The ICC agreement reliability scores measuring differences between the distractor and non-distractor scenarios on total score were moderate (0.502). For the specific categories, the ICC scores varied between slight and moderate (see Table 3).

**Table 3:** Interrater reliability of measuring differences between research conditions for the categories of the ANTS system

Categories	ICC difference
Task Management	0.438
Team Working	0.072
Situation Awareness	0.417
Decision Making	0.576

### Usability

Both raters indicated that they considered the scores obtained by the ANTS system to provide a good reflection of the NTS of the physicians. The ANTS system was judged to address all key NTS behaviors, and although some of the elements were considered to overlap to some extent (i.e., checking the quality of a task performed by a team member could attribute to “Using Authority and Assertiveness,” “Assessing Capabilities,” and “Re-evaluation”), they were not considered to be redundant. The categories were considered to be observable and easy to rate (see Table 4). For rating the elements, they considered that consensus on a list of good and a poor behavior was prerequisite. With the list of good and poor behaviors, the elements could be rated, with the exception of Decision Making, which was considered difficult in the scenarios that were used in the present study.

**Table 4:** Observability and difficulty scores of the elements on a four-point scale as judged by the raters in this study

Categories	Elements	Observability^a^		Difficulty^b^	
		Rater 1	Rater 2	Rater 1	Rater 2
Task Management	Planning and Preparing	3	3	4	3
	Prioritizing	4	4	4	4
	Providing and Maintaining Standards	4	4	3	4
	Identifying and Utilizing Resources	3	4	3	4
Team Working	Coordinating Activities with Team	4	4	4	4
	Exchanging Information	4	4	4	3
	Using Authority and Assertiveness	4	4	4	4
	Assessing Capabilities	4	3	4	4
	Supporting Others	4	4	4	4
Situation Awareness	Gathering Information	4	3	4	3
	Recognizing and Understanding	3	3	2	3
	Anticipating	3	3	3	3
Decision Making	Identifying Options	3	2	2	3
	Balancing Risks and Selecting Options	2	2	1	3
	Re-evaluating	3	3	2	3

^a^The observability of the elements: 1 = poor, 2 = marginal, 3 = satisfactory, 4 = good

^b^The difficulty to score the elements: 1 = very difficult, 2 difficult, 3 = average, 4 = easy

## Discussion

### Main findings and interpretation

The interrater reliability of the ANTS system was substantial for our two raters. The ICC of the different categories and elements were fair to substantial. The reliability to measure differences between conditions was moderate. The raters judged the ANTS system as a usable behavioral marker system and considered the ANTS score as representative of the NTS of the physicians in the videos.

This study showed that the ANTS system has reasonable psychometric properties in our study. While higher reliability scores would be desirable (>0.7), we feel that with an overall substantial reliability on the evaluation of complex behavior like NTS, the ANTS can be also recommended for research situations. Our study broadens the spectrum of the ANTS system from an educational tool to a tool that is used to assess the NTS for research purposes. The expected differences in NTS scores for the non-distractor and distractor conditions were revealed by the ANTS, and the ICC scores of measuring these differences between conditions show that differences in NTS scores between conditions can be found with a reasonable reliability, which also suggests a reasonable validity of the ANTS system.

The reliability of the ANTS system in our study is sufficient but lower than the reliability of some other studies on NTS [14, 18, 19]. There are several reasons that can explain the lower reliability values. First, previous studies only rated the highly observable elements of the rating system, while in this study, the reliability was calculated based on all elements of the ANTS system, including the elements that were not easy to observe. While for educational purposes it is reasonable not to rate the elements that have a poor observability in a certain scenario, in a research setting, it is necessary to compare the scores between research conditions and participants. Therefore, the same elements should be rated for all participants. Since the elements with the lowest ICC values are also considered to be the most difficult and the least observable aspects in the usability measures, i.e., Assessing Capabilities, this might explain the generally lower ICC scores. Secondly, the relatively low reliability and the poor observability and difficulty for the Decision Making elements can be explained by the lack of decision making behaviors in the scenarios used in this study.

Based on this study, there is no reason to revise the ANTS system. The categories and most elements of the ANTS had a moderate reliability, and the elements with only a fair reliability were not the behaviors that are typically present in a resuscitation scenario. Specifically, a resuscitation scenario is mainly about performing the elements of the guidelines in a timely manner and in the right order, rather than a decision making process that involves identifying and evaluating options. In other scenarios, e.g., a problematic intubation may involve many decision making behaviors but other ANTS elements may be harder to observe. We recommend not to rate the elements that are insufficiently represented in the scenario of interest. A pilot study to identify the ANTS elements that are applicable in the scenario at hand is recommended to ensure sufficient interrater reliability. In order to allow for comparison of participants and different experimental conditions in a research setting, only these elements that are applicable to the scenario should be assessed in the participants.

The usability measures suggest that the ANTS system is usable for research purposes. The raters did indicate that creating a list of poor and good behaviors was essential. It is therefore recommended that a list of scenario-specific poor and good behaviors is developed. However, these results should be interpreted with caution given that only two raters were involved.

### Strengths and limitations

This study is of value because it shows that the ANTS system is suitable to measure the NTS in a research setting where all elements of the NTS are part of the evaluation, particularly because differences between research conditions can be reliably revealed. Additionally, this study shows that the ANTS system is usable and that the ANTS handbook provides sufficient information to the raters to use the ANTS system.

There are several limitations of this study. First, the study is based on two raters, and therefore, the results lack generalizability. This is especially the case for the usability data. Second, the results of this study are based on 60 resuscitation scenarios and therefore might not be generalizable to other scenarios. Third, the training that the raters received was not very extensive. This shows that the reliability and usability of the ANTS system is sufficient even with little training, which is in contrast to a study by Graham et al. [15]. If a more standardized training for the ANTS system would be developed, the reliability might have been better. Furthermore, the raters showed a good consistency (high correlation) but differed in the mean value of the scores. An ICC consistency measure would therefore have been higher than the ICC agreement that we used.

## Conclusions

The ANTS system seems to be a reliable system to use for research purposes even when poorly observable elements were included in the score. The ANTS system can also reliably measure differences between research conditions.

The usability was judged to be good, although this result should be interpreted with caution given that it was based on the scores of two raters. For future studies, it is recommended to include the behavioral elements that are sufficiently represented in the scenario.

## Additional file

Additional file 1: Usability questionnaire: The two raters who participated in this study both filled out the questionnaire on the usability of the ANTS system. (PDF 120 kb)

## References

[1] Fletcher G, McGeorge P, Flin R, Glavin R, Maran N (2002). The role of non-technical skills in anaesthesia: a review of current literature. Br J Anaesth.

[2] Gaba D, Fish K, Howard S (1994). Crisis management in anesthesiology.

[3] Flin R, O’Connor P, Crichton M. Safety at the sharp end: a guide to non-technical skills. Hampshire England: Ashgate Publishing Limited; 2008.

[4] Byrne A, Sellen A, Jones G (2002). Effect of videotape feedback on anaesthetists’ performance while managing simulated anaesthetic crises: a multicentre study. Anaesthesia.

[5] Flin R, Yule S, Paterson-Brown S, Maran N, Rowley D, Youngson G (2007). Teaching surgeons about non-technical skills. Surgeon.

[6] Mishra A, Catchpole K, Dale T, McCulloch P (2008). The influence of non-technical performance on technical outcome in laparoscopic cholecystectomy. Surg Endosc.

[7] Fletcher G, Flin R, McGeorge P, Glavin R, Maran N, Patey R (2004). Rating non-technical skills: developing a behavioural marker system for use in anaesthesia. Cogn Tech Work.

[8] Yule S, Flin R, Paterson-Brown S, Maran N, Rowley D (2006). Development of a rating system for surgeons’ non-technical skills. Med Educ.

[9] Flin R, Martin L, Goeters K (2003). Development of the NOTECHS (non-technical skills) system for assessing pilots’ CRM skills. Hum Factors Aerospace Saf.

[10] Gaba D, Howard S, Flanagan B, Smith B, Fish K, Botney R (1998). Assessment of clinical performance during simulated crisis using both technical and behavioral ratings. Anesthesiol.

[11] Cooper S, Endacott R, Cant R (2010). Measuring non-technical skills in medical emergency care: a review of assessment measures. Open Acces Emerg Med.

[12] Yee B, Naik V, Joo H (2005). Nontechnical skills in anesthesia crisis management with repeated exposure to simulation-based education. Anesthesiol.

[13] Flin R, Patey R, Glavin R, Maran N (2010). Anaesthetists’ Non-Technical Skills. Br J Anaesth.

[14] Fletcher G, Flin R, McGeorge P, Glavin R, Maran N, Patey R (2003). Anaesthetists’ Non-Technical Skills (ANTS): evaluation of a behavioural marker system. Br J Anaesth.

[15] Graham J, Hocking G, Giles E (2010). Anaesthesia Non-Technical Skills: can anaesthetists be trained to reliably use this behavioural marker system in 1 day?. Br J Anaesth.

[16] Aberdeen Uo. Framework for observing and rating Anaesthetists’ Non-Technical Skills; Anaesthetists’ Non-Technical Skills (ANTS) System Handbook v 1.0. http://www.abdn.ac.uk/iprc/ants/. 2003.

[17] Landis J, Koch G (1977). The measurement of observer agreement for categorical data. Biometrics.

[18] Mishra A, Catchpole K, McCulloch P (2009). The Oxford NOTECHS System: reliability and validity of a tool for measuring teamwork behaviour in the operating theatre. Qual Saf Health Care.

[19] Yule S, Flin R, Maran N, Rowley D, Youngson G, Paterson-Brown S (2008). Surgeons’ non-technical skills in the operating room: reliability testing of the NOTSS behavior rating system. World J Surg.

