# Supplementary material for: The reliability and usability of the Anesthesiologists’ Non-Technical Skills (ANTS) system in simulation research
Source: Adv Simul (Lond). 2016 Jun 8;1:18. doi: 10.1186/s41077-016-0013-2 (PMC5806494; doi:10.1186/s41077-016-0013-2)
Supplement: Supplementary file 1 — Usability questionnaire: The two raters who participated in this study both filled out the questionnaire on the usability of the ANTS system. (PDF 120 kb) [file 41077_2016_13_MOESM1_ESM.pdf]

1. Did it address the key NTS behaviours displayed?

Yes No

Comment……………………………………………..

2. Do you think any elements or categories are missing?

Yes No

Comment……………………………………………..

3. Do you think any elements or categories are superfluous?

Yes No

Comment……………………………………………..

4. How easy was it to relate behaviours to categories?

Average to easy Difficult to very difficult?

Comment:………………………………………………..

5. Were all categories observable in the videos?

Yes No

Comment………………………………………………..

Please specify the observability and difficulty per category:

1=poor, 2= marginal, 3= average, 4= good

Categories	***Observability***	***Difficulty***
**Task Management**		
**Team Working**		
**Situation Awareness**		
**Decision Making**		

6. How easy was it to relate behaviours to elements?

Average to easy Difficult to very difficult?

Comment:……………………………………………………..

7. Were all categories observable in the videos?

Yes No

Comment………………………………………………..

Please specify for each element the observability and difficulty:

1=poor, 2= marginal, 3= average, 4= good

Categories	***Elements***	***Observability***	***Difficulty***
**Task Management**	Planning & preparing		
	Prioritising		
	Providing & maintaining standards		
	Identifying and utilising resources		
**Team Working**	Co-ordinating activities with team		
	Exchanging information		
	Using authority & assertiveness		
	Assessing capabilities		
	Supporting others		
**Situation Awareness**	Gathering information		
	Recognising & understanding		
	Anticipating		
**Decision Making**	Identifying options		
	Balancing risks & selecting options		
	Re-evaluating		

8. Do you think the rating scale gave you enough flexibility to rate the performance levels (or would you prefer a longer or shorter scale)?

Yes No

If no: longer, shorter or other comment? ……………………………………………………….

9. Do you think the final score reflects the non technical performance correctly?

Yes No

Comment……………………………………………
